# Cell sorters see things more clearly now

**DOI:** 10.15252/msb.202211254

**Published:** 2023-02-13

**Authors:** Daniel Schraivogel, Lars M Steinmetz

**Affiliations:** ^1^ Genome Biology Unit European Molecular Biology Laboratory (EMBL) Heidelberg Germany; ^2^ Department of Genetics Stanford University School of Medicine Stanford CA USA; ^3^ Stanford Genome Technology Center Palo Alto CA USA

**Keywords:** Biotechnology & Synthetic Biology

## Abstract

Microscopy and fluorescence‐activated cell sorting (FACS) are two of the most important tools for single‐cell phenotyping in basic and biomedical research. Microscopy provides high‐resolution snapshots of cell morphology and the inner workings of cells, while FACS isolates thousands of cells per second using simple parameters, such as the intensity of fluorescent protein labels. Recent technologies are now combining both methods to enable the fast isolation of cells with microscopic phenotypes of interest, thereby bridging a long‐standing gap in the life sciences. In this Commentary, we discuss the technical advancements made by image‐enabled cell sorting and highlight novel experimental strategies in functional genomics and single‐cell research.

## Graphical abstract

This Commentary discusses the technical advancements made by image‐enabled cell sorting and highlights novel experimental strategies in functional genomics and single‐cell research.

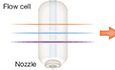

## Removing a technological blind spot

**Figure 1:**
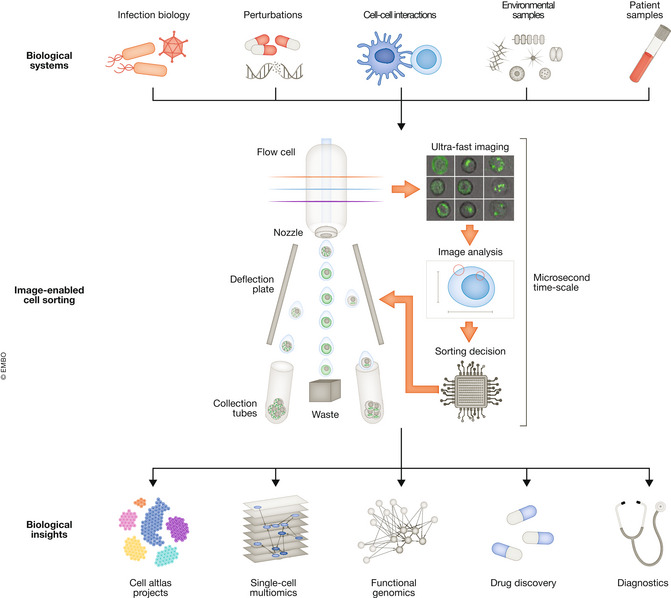
Combining imaging with cell sorting enables new experimental strategies across the life science disciplines Examples of prime applications for image‐enabled cell sorting. *Top*: Image‐enabled cell sorting isolates cells from multiple sample sources, such as *in vitro* model systems that were genetically perturbed, treated with drugs, or infected with bacteria or viruses. In addition, dissociated tissues, patient biopsy material, and environmental samples can be used to isolate spatial phenotypes of interest. *Middle*: Separation according to spatial microscopic phenotypes by classifying events according to phenotypic measurements or machine learning‐based methods. *Bottom*: Sorting using image information identifies novel cell types and states for cell atlas projects, is used for single‐cell multi‐omics (see main text), or enables high‐speed functional genomic screening. Cells with specific phenotypic properties, such as production cell lines that generate drugs, or highly active T‐cells that preferentially interact with antigen‐presenting cells, are isolated. Information from images will be used for the fast diagnosis of diseases.

To understand the biology and function of individual cells within complex cell populations, cells with specific properties often need to be isolated. The ability to physically isolate cells unlocks downstream analyses for a closer characterization of cells of interest: For example, isolated cells can be cultivated to characterize cellular functions, or used for omics assays, such as genome sequencing, transcriptomics, and proteomics, to uncover the molecular mechanisms underlying cellular traits. The workhorse in cell isolation in the life sciences is FACS, which was invented in the 1960s for the purpose of enriching viable cells. Since then, the information that could be acquired from each cell constantly increased, allowing high‐dimensional sorting using up to 40 cellular properties and sorting speeds over 10,000 cells per second. Nonetheless, FACS is like looking at an artwork through tinted glass: Only simple morphological parameters such as approximate cell size and the expression levels of labeled proteins can be used for separation. However, a large part of cellular phenotypes is not associated with changes in protein abundance, but rather with spatial phenotypic changes, such as changes in the intracellular localization of proteins. For example, transcription factors can be tethered to the cytoplasm rendering them inactive, and the regulated transition into the nucleus triggered by signaling pathways provides the transcription factor access to the genome. Similarly, mutant proteins frequently mislocalize in disease, such as proteins that aggregate in neurodegenerative diseases and heart diseases. Traditional FACS is blind to such phenotypes. Enriching cells with spatial phenotypes of interest provides a deeper understanding of the causes and consequences of cellular morphology and protein localization and holds promise for identifying novel biomarkers and drug targets (Fig 1).

The process of isolating cells with spatial phenotypes of interest can be subdivided into three steps: cell imaging, cell classification, and cell isolation. Micromanipulation methods, such as laser capture microdissection, were among the first methods to isolate cells with specific microscopic phenotypes. These methods collect cells using a microcapillary or focused laser beam under a microscope. Alternatively, photoactivatable proteins can be used to mark cells of interest under a microscope by means of photoactivation, followed by isolation of the tagged cells using FACS. Although they have higher throughput than micromanipulation, especially if combined with automation and machine learning for cell classification, these methods are still limited in throughput (> 1 week for genome‐scale experiments) and come with delays between cell classification and isolation. Adding imaging capabilities to high‐throughput cell sorting devices such as FACS holds promise for increasing throughput by orders of magnitude, thereby solving a massive technological gap in the life sciences.

Cell sorting platforms with imaging capabilities have been developed and can be classified into microfluidic cell sorters and droplet‐based flow cytometric sorters. FACS is an imaging‐free example of the latter. Microfluidic systems are generally slower (< 1,000 cells per second), while FACS works at least one order of magnitude faster (> 10,000 cells per second). The main reason for the differences in speed is how cells are isolated: In FACS, cells contained in liquid droplets are separated by electrically charging a droplet followed by deflection in an electrical field, whereas valves need to be opened and closed to channel a cell in a microfluidic sorter. Developing cell sorting technologies with imaging capabilities is complicated for several reasons. First, in traditional FACS, cells pass the laser interrogation point at a high speed of more than 1 m/s, exemplifying the need for specialized imaging technologies that generate blur‐free images of fast‐flowing cells. We recently introduced image‐enabled cell sorting (ICS) together with BD Biosciences, which combines FACS with Fluorescence Imaging using Radiofrequency‐tagged Emission (FIRE; Schraivogel *et al*, 2022). FIRE is an imaging technology based on a laser beam that is split into multiple beams, which are modulated at different radio frequencies, allowing to obtain data of many pixels simultaneously. Another method, termed image‐activated cell sorting II (IACS II), uses Virtual Freezing Fluorescence Imaging (VIFFI), an optomechanical imaging method, in a microfluidic device (Isozaki *et al*, 2020). Other systems, such as the DeepCell platform, use a high‐speed camera for brightfield imaging in a microfluidic stream (preprint: Salek *et al*, 2022). Altogether, these methods allow blur‐free imaging of fast‐flowing cells, and most technologies obtain both label‐free (e.g., brightfield) and fluorescence images. Second, the short time available between signal acquisition and sorting decision (400 μs to 30 ms depending on the technology) requires low‐latency electronics for image analysis. These methods often make use of field‐programmable gate arrays (FPGAs) or graphic processing units (GPUs) for ultra‐fast image reconstruction, analysis, and classification. Image analysis and classification can be based on predefined user‐understandable parameters that are calculated from images in real time (such as in ICS). Alternatively, parameter‐free machine intelligence can be used for cell classification (such as neural networks in IACS). Other image‐sorting platforms combine the advantages of both classification strategies (such as DeepCell). While user‐defined parameters allow immediate sorting based on the human understanding of a spatial phenotype, parameter‐free machine learning maximizes compatibility with difficult‐to‐distinguish phenotypes but often depends on large annotated datasets for training.

## Speeding up imaging‐based screens

High‐throughput functional genomics aims to understand genome function by reading, writing, and editing genomes across scales. Acquiring knowledge through experimental perturbation, rather than genetic association, identifies causal disease variants, novel drug targets, and biomarkers. The field currently experiences a revolution driven by the adaption of single‐cell technologies and CRISPR/Cas9 for pooled screening applications. Single‐cell technologies, such as scRNA‐seq, FACS, and ICS, increase the complexity of pooled genetic screening readouts, compared with that of simple readouts such as cellular growth or viability. Imaging‐based readouts have only recently become available for pooled screening approaches, due to the difficulty of extracting microscopic phenotype and perturbation genotype from the same cell. We and others solved this issue by combining pooled CRISPR/Cas9 screens with different imaging technologies and varying strategies for perturbation genotyping. For example, ICS was used to enrich cells with microscopic phenotypes of interest, followed by the extraction of genomic DNA from sorted cells and sequencing of genome‐integrated barcodes. These barcodes report on the perturbation identity of each cell and identify genes that regulate the localization of a protein of interest (Schraivogel *et al*, 2022). ICS also enables isolating single cells for downstream single‐cell omics analysis and, consequently, pairing images with omics readouts on a per‐cell basis. Other methods for imaging‐based functional genomics make use of traditional fluorescence microscopy technology, followed by sequencing of perturbation‐associated barcodes *in situ* (i.e., sequencing under the microscope without physically isolating cells; Feldman *et al*, 2019). Alternatively, multiplexed fluorescence *in situ* hybridization identifies perturbation barcodes using microscopy (Wang *et al*, 2019). Last but not least, cells that express a photoactivatable protein allow tagging cells of interest under a microscope by means of photoactivation, followed by FACS to isolate activated cells and sequencing of perturbation barcodes (Kanfer *et al*, 2021; Yan *et al*, 2021). All three methods differ in throughput, technical complexity, and compatibility with different phenotypes. The throughput of ICS enables genome‐wide screens with imaging readouts in hours or days, compared with weeks or months with other pooled methods or arrayed microscopic screens. Microscopy‐based methods, however, have higher resolution, making a larger phenotypic space available to pooled screening, but with the limitation of reduced speed. In addition, methods based on photoactivation are incompatible with cell fixation and therefore cannot capture short‐lived dynamic phenotypes.

ICS makes genome‐wide microscopy‐based screens available to nonspecialized laboratories due to its ease of use and high speed. Nonetheless, to meet the full complexity of genomes, we ultimately want to unlock screens that are far more complex than targeting all 20,000 human protein‐coding genes. For example, combinatorial perturbations that target combinations of genes or genetic loci in the same cell can identify genetic interactions. This is particularly interesting for noncoding regulatory regions such as enhancers, which often work cooperatively to regulate protein‐coding gene expression. To saturate the noncoding space and to enable combinatorial perturbations, such deep dives into the complexity of the genome will not only depend on scalable, affordable, and sensitive readouts but also intricate perturbation strategies using large CRISPR libraries with maximized effect sizes. Novel ultracompact CRISPR libraries with improved gRNA design and multi‐gRNA libraries significantly broaden the scope of perturbation screens. Combined with fast imaging and sorting technologies such as ICS, these novel library designs will decrease run time for a genome‐wide coding screen down to a few hours. Such developments are critical to work towards the goal of assigning functions and phenotypes to every coding and noncoding region in the human genome. Combined with precision genome editing tools, such phenotypes will even be assigned at single nucleotide resolution, to ultimately assign functions at single nucleotide resolution.

## Future developments

Cells, even those of the same cell type, show a remarkable diversity of phenotypes, including differential gene expression, protein localization, and cellular morphology. Our definition of cell types and states is largely driven by single‐cell technologies that measure cellular morphology (e.g., microscopic phenotypes), protein expression (e.g., surface markers), and transcriptomic state (e.g., scRNA‐seq data). Cell sorting technologies with imaging capabilities will be helpful in identifying novel cell types according to spatial phenotypes and isolating them for functional characterization. Combining ICS with other omics assays, such as protein‐ (e.g., CITE‐seq, scMS), transcript‐ (e.g., scRNA‐seq), and genome‐centric (e.g., scATAC‐seq, Strand‐seq) readouts, will further increase the resolution of single‐cell profiling experiments. Strikingly, recent studies demonstrated machine learning algorithms that can estimate genotype, cell‐cycle state, and transcriptomic state directly from low‐resolution brightfield imaging data (Chlis *et al*, 2020). The impact of these findings is substantial: For example, cell types and states will soon be identified and quantified from dissociated patient biopsies for diagnostic purposes by imaging rather than sequencing at no cost and within minutes. Upon identification, we will be able to isolate cells of interest from primary patient material for downstream functional assays, such as drug sensitivity tests that benchmark possible therapy strategies and identify the optimal one for a given patient. Traditional FACS does not provide the rich information contained in microscopic images to accurately predict cell states. At the same time, scRNA‐seq assays are destructive and time‐consuming and do not scale to large patient numbers that need to be analyzed on demand. In addition to diagnostic applications, multiomic readouts from single cells will impact the field of functional genomics. Pooled screens with single‐cell readouts assay complex cellular phenotypes at scale and provide a deep understanding of genome regulation and gene expression in health and disease. For example, a combined readout of single‐cell imaging and scRNA‐seq will connect cellular genotypes and phenotypes via changes in gene expression, thereby providing insights into the mode‐of‐action (transcriptome) and consequences (cellular phenotype) of a perturbation at the same time. A prime application is the characterization of disease‐associated genetic variation, including noncoding genetic variants for which we cannot predict a mode‐of‐action and that have barely been connected to cellular phenotypes.

Although technologies such as ICS, IACS, and Deepcell provide lower resolution imaging data than traditional microscopy, the information content from low‐resolution imaging data is large, and especially powerful if retrieved from many cells and multiple imaging channels. For example, 16‐bit images (16 possible values per pixel) consisting of 400 pixels within a cell across seven imaging channels can in principle distinguish 2^44,800 possible states. This exponentially expands as new imaging modalities are added, such as additional colors, or the resolution increases. For example, images with a greater number of fluorescent channels would allow for cell painting studies. In cell painting, different cellular organelles, such as the nucleus, mitochondria, and cytoskeleton, are fluorescently labeled, which significantly increases the phenotypic space available for cell classification. Further increasing the resolution to that of traditional fluorescence microscopy, or combining cell sorting with 3D imaging, will depend on future technological advancements both on the imaging and data processing side. These advancements include faster image analysis, computer guided cell classification, and increasing the detection sensitivity at ultra‐short exposure time. Such complex imaging modes ultimately unlock high‐speed image‐enabled sorting of highly complex single‐cell phenotypes, cell complexes, or 3D organoids.

## Disclosure and competing interests statement

The authors declare that they have no conflict of interest.
